# Self-Emulsifying Drug Delivery Systems (SEDDS): Measuring Energy Dynamics to Determine Thermodynamic and Kinetic Stability

**DOI:** 10.3390/ph15091064

**Published:** 2022-08-26

**Authors:** Fiza Ur Rehman, Arshad Farid, Shefaat Ullah Shah, Muhammad Junaid Dar, Asim Ur Rehman, Naveed Ahmed, Sheikh Abdur Rashid, Irfan Shaukat, Muddaser Shah, Ghadeer M. Albadrani, Mohamed Kamel, Ahmed E. Altyar, Mohamed M. Abdel-Daim, Kifayat Ullah Shah

**Affiliations:** 1 Department of Pharmacy, Faculty of Biological Sciences, Quaid-i-Azam University, Islamabad 45320, Pakistan; 2 Gomal Center of Biochemistry and Biotechnology, Gomal University, Dera Ismail Khan 29050, Pakistan; 3 Skin/Regenerative Medicine and Drug Delivery Research, GCPS, Faculty of Pharmacy, Gomal University, Dera Ismail Khan 29050, Pakistan; 4 Department of Biochemistry, University of Narowal, Narowal 51600, Pakistan; 5 Department of Botany, Abdul Wali Khan University Mardan, Mardan 23200, Pakistan; 6 Natural and Medical Sciences Research Center, University of Nizwa, P.O. Box 33, Birkat Al Mauz, Nizwa 616, Oman; 7 Department of Biology, College of Science, Princess Nourah bint Abdulrahman University, P.O. Box 84428, Riyadh 11671, Saudi Arabia; 8 Department of Medicine and Infectious Diseases, Faculty of Veterinary Medicine, Cairo University, Giza 12211, Egypt; 9 Department of Pharmacy Practice, Faculty of Pharmacy, King Abdulaziz University, Jeddah 21589, Saudi Arabia; 10 Department of Pharmaceutical Sciences, Pharmacy Program, Batterjee Medical College, P.O. Box 6231, Jeddah 21442, Saudi Arabia; 11 Pharmacology Department, Faculty of Veterinary Medicine, Suez Canal University, Ismailia 41522, Egypt

**Keywords:** thermodynamic stability, kinetic stability, lipidic formulations, microemulsions, nanoemulsions, self-emulsifying drug delivery system (SEDDS), finasteride

## Abstract

This research was designed to identify thermodynamically and kinetically stable lipidic self-emulsifying formulations through simple energy dynamics in addition to highlighting and clarifying common ambiguities in the literature in this regard. Proposing a model study, this research shows how most of the professed energetically stable systems are actually energetically unstable, subjected to indiscriminate and false characterization, leading to significant effects for their pharmaceutical applications. A self-emulsifying drug delivery system (SEDDS) was developed and then solidified (S-SEDDS) using a model drug finasteride. Physical nature of SEDDS was identified by measuring simple dynamics which showed that the developed dispersion was thermodynamically unstable. An in vivo study of albino rats showed a three-fold enhanced bioavailability of model drug with SEDDS as compared to the commercial tablets. The study concluded that measuring simple energy dynamics through inherent properties can distinguish between thermodynamically stable and unstable lipidic systems. It might lead to correct identification of a specific lipidic formulation and the application of appropriate characterization techniques accordingly. Future research strategies include improving their pharmaceutical applications and understanding the basic differences in their natures.

## 1. Introduction

Self-emulsifying drug delivery systems (SEDDS) have earned a reputation as a vital strategy to improve bioavailability of poorly aqueous soluble drugs belonging to BCS (Biopharmaceutical Classification System) classes II and IV [1,2]. The main interest of these nano droplet-generating systems is their stability and greater potential in pharmaceutical applications with an easy industrial scale-up due to simple formulation processes [3,4,5,6]. These systems are described as self-nanoemulsifying (SNEDDS) and self-microemulsifying drug delivery systems (SMEDDS) as per their ability to generate nanoemulsion (NE) and microemulsion (ME), respectively [7,8]. NE are conventional emulsions with a droplet size of ∼300 nm, whereas ME resemble swollen micelles with droplet size preferably below 100 nm, in which the oil phase is dispersed inside the hydrophobic core of surfactant micelles [9,10,11]. The nature of ME is fundamentally identical to micelles i.e., they are developed by amphiphiles at critical micelle concentration (CMC) and incorporate the dispersed phase at their core. However, the droplets in microemulsions contain much more dispersed liquid than in swollen micelles. Therefore, despite all the differences, ME resemble micelles in their basic nature and differ from true emulsions. There are a lot of similarities between ME and NE e.g., (a) similar basic components (nonionic or ionic amphiphiles, oil and co-surfactant); (b) both can be formulated through low energy methods (spontaneous generation); (c) both require little external energy to support mass transport and to remove barriers of kinetic energy; (d) common nanometric size range of droplets; (e) similar apparent droplet structure (hydrophilic and hydrophobic parts arranged according to the continuous phase) and (f) both the colloidal dispersions have been described in the literature with similar terminology i.e., ME and NE, incorrectly implying that the two systems differ only in particle size range [11,12,13,14]. Similar manufacturing processes, close molecular and visual aspects, and use of occasional inappropriate terminology e.g., NE described in the literature as mini, micro, submicron, or ultrafine emulsions often lead to confusion between the two systems, resulting in a false interpretation. Moreover, under specific temperature ranges and concentrations, microemulsions have spherical nano-sized droplets similar to those of nanoemulsions, further perpetuating the mater [15].

NE are thermodynamically unstable but kinetically stable dispersions which can be prepared through high energy as well as low energy methods, while ME are kinetically unstable but thermodynamically stable dispersions, which are generated spontaneously through low energy methods [16,17]. The former has spherical droplets which have varying sizes (lying in a close range), whereas the latter show specifically similar size droplets which may or may not be spherical in shape. NE require a specific order of mixing of components but ME would be the same whatever the order of mixing [9,13]. A plethora of publications report the fabrication, characterization and in vitro or in vivo evaluation of SMEDDS [18,19,20,21]. First, physicochemical properties (droplet size distribution, shape, effect of dilution, and change in temperature) of most of these formulations imply that they are SNEDDS not SMEDDS. Therefore, methods that authors previously reported for the characterization of SMEDDS raise questions, e.g., ternary phase diagrams and dilution prior to particle size analysis are not suitable for SMEDDS. Second, the name SMEDDS itself seems illogical since microemulsions are truly spontaneous formulations by nature. It appears that the droplets in NE are smaller than those in ME but in actuality, the opposite is true [22,23]. This mix-up leads to the significant effects in pharmaceutical applications of such nanomedicines. For instance, generally ME are more stable than NE due to thermodynamic stability but temperature changes during shelf-life may affect ME more adversely than NE [24]. Stability of NE depends upon the extent of its kinetic stability i.e., higher the energy barrier between separated individual components and NE state, greater is the kinetic stability and consequently NE are said to have greater stability. Even if the storage conditions stay same, because of its thermodynamic instability, NE would eventually break down into its components, sooner or later, depending on its kinetic stability, whereas as ME is thermodynamically stable, it would remain same unless its temperature, pressure, or composition is altered during its use, storage, or manufacturing [12,15].

Finasteride (FDE), a type-II steroid and 5-alpha–reductase specific competitive inhibitor, belongs to BCS class II and chemically is N-(1,1-dimethylethyl)-3-oxo-4-aza-5-alpha-androst-1-ene-17-Beta-carboxamide. It is used clinically to treat androgenetic alopecia, benign prostatic hyperplasia (BPH), and prostate cancer [25,26]. Finasteride is a highly lipophilic drug with log P value 3.03. A daily dose of 1 mg and 5 mg is used for androgenetic alopecia and benign prostatic hyperplasia [27]. Finasteride is metabolized in the liver by CYP3A4 enzymes and is a substrate for P-glycoprotein efflux transporters in the gastrointestinal tract and hence low bioavailability (63%) [28]. The stated physicochemical properties of the finasterid, suggests that it is a candidate for solubility enhancement through SEDDS.

The aim of this research was to set forth a model study in order to identify stable emulsion. Thermodynamically different systems were differentiated on the basis of their intrinsic properties through simple differentiation tests. The SEDDS, using finasteride as a model drug, was developed and characterized through novel and simple differentiation tests for the determination of emulsion stability. Moreover, in vivo pharmacokinetics was evaluated to see the oral delivery potential of SEDDS. This study might be of significant importance in describing the true nature of a lipidic system and assigning a SEEDS formulation as SNEDDS or SMEDDS.

## 2. Results and Discussion

### 2.1. Selection of Oil, Surfactant and Cosurfactant

**Figure 1:**
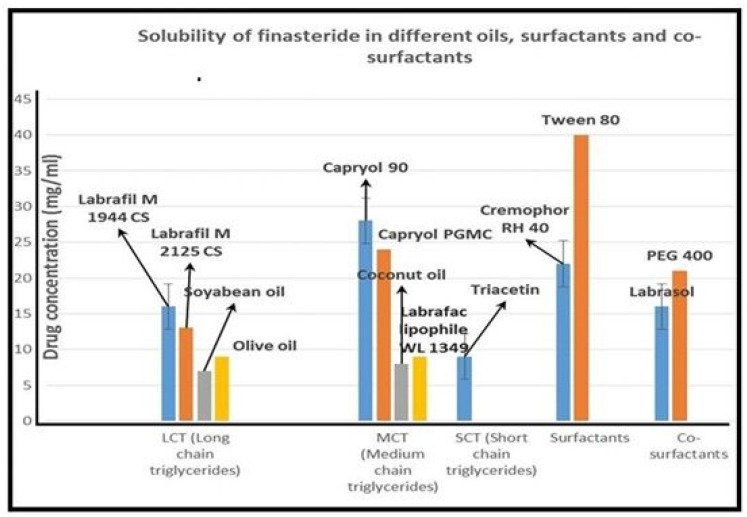
Solubility of finasteride in different oils, surfactants and co-surfactants.

The saturation solubility of FDE in different vehicles is shown in Figure 1. Among all the selected oils, Labrafil M 1944CS and Capryol 90 showed the highest solubility for FDE and were selected for the future studies. Non-ionic surfactants are generally considered safe for oral ingestion. For the present study, the selection of surfactant was based on their solubilizing capacity and emulsification ability. As shown in Table 1, Tween 80 has the highest solubilizing capacities for finasteride, whereas Cremophore RH 40 showed considerable solubilizing capacity. Therefore, both these non-ionic surfactants were further tested for their ability to emulsify the screened oils. Their emulsification ability was determined by evaluating self-emulsification time and percentage transmittance of the resulted dispersion. Cremophore RH 40 gave better transmittance and shorter Self-emulsification time with both the oils as compared to Tween 80. Moreover, percentage transmittance of the emulsion prepared using Capryol 90 and Cremophore RH 40 was the highest. Thus, Cremophore RH 40 and Capryol 90 were chosen for further investigation as surfactant and oil, respectively. Though PEG 400 and Labrasol showed similar transmittance of the formulated emulsion, PEG 400 has greater ability to solubilize finasteride (Figure 1) compared to Labrasol; therefore, it was selected as a co-surfactant to prepares stable emulsion with highest finasteride pay load.

### 2.2. Ternary Phase Diagram and Features of SEDDS

**Figure 2:**
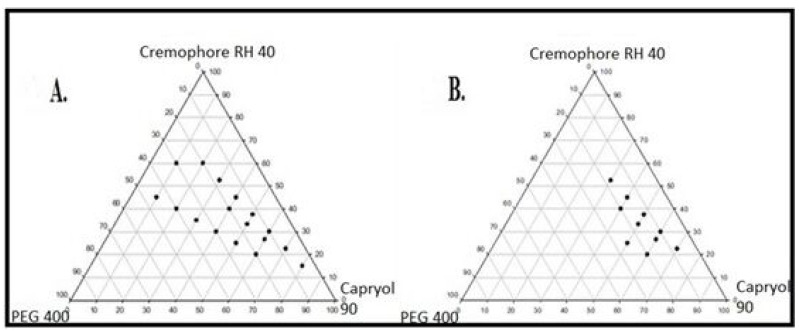
Pseudo ternary phase diagrams of SNEDDS (**A**) Blank formulations, (**B**) finasteride loaded formulations.

Ternary phase diagrams optimized the concentrations of oil (Capryol 90), surfactant (Cremophore RH 40) and co-surfactant (PEG 400) for the SEDDS formulation. The self-emulsifying region was identified and is shown in Figure 2A,B. It is noted that when the surfactant to co-surfactant ratio was (3:1), self-emulsifying region was the largest among all the tested SEDDS formulations. The results in Table 2 show that out of several SEDDS formulations prepared, formulation 21 (oil, surfactant, and co-surfactant, 30:52.5:17.5, respectively) showed the most ideal features with 94% transmittance: the smallest droplet size of 180 nm and maximum loading of finasteride. When subjected to dilution with mild agitation, SEDDS should self-emulsify rapidly and completely [9,29]. Self-emulsifying efficiency of SEDDS formulations with different excipient ratios was observed by noting the time for self-emulsification as shown in Table 2. Formulation 21 was the quickest in self-emulsification and producing SEDDS.

### 2.3. Optimization of Finasteride SEDDS in Liquid Form (L-SEDDS)

The final formulation was chosen on the basis of percentage transmittance, FDE solubilizing capacity, and self-emulsification time. Table 2 shows that formulation 21 has the highest percentage transmittance (94%), self-emulsification time of only 10 s and maximum solubilizing capacity for finasteride (22 mg/mL). Therefore, formulation 21 was selected as optimized FDE loaded L-SEDDS (SEEDS in liquid form prior to adsorption on carrier for solidification as tablets) formulation and was subjected to further characterization.

### 2.4. Differentiation between Nanoemulsions and Microemulsions

**Figure 3:**
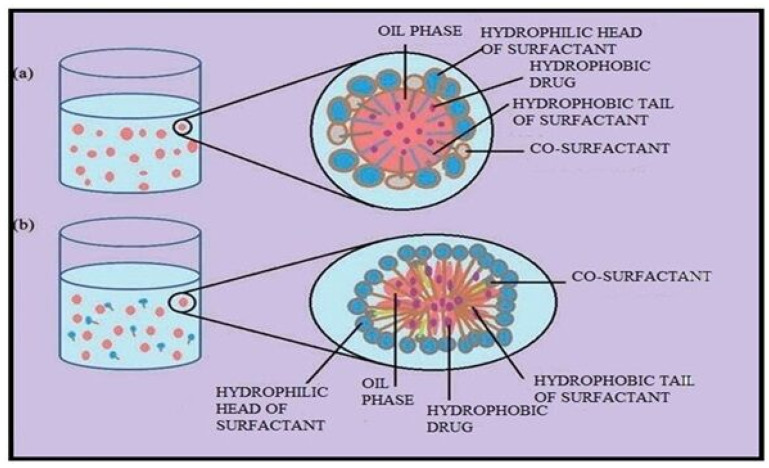
Schematic diagram showing the structures of (**a**) Nanoemulsion droplet and (**b**) Microemulsion droplet. Both are made up of surfactant, co-surfactant, and oil. Nanoemulsion droplets are spherical and show polydispersity in their size (**a**) whereas microemulsion droplets can be of a shape that is not necessarily spherical, and all droplets are of same diameter and shape (**b**). Reproduced with the consent of authors. Adapted from [2].

NE have greater free energies as compared to their individual components, hence, referred to as thermodynamically unstable systems which are at non-equilibrium stage. On the contrary, ME are thermodynamically stable with low free energies when compared to their individual components [17,30]. The former are kinetically stable nano systems because of very slow degradation kinetics (reaching several months), while the later are prone to degradation due to faster kinetics of degradation. The ME need higher surfactant amount in general (not only surfactant-oil ratio) because this is the condition to reach the ultralow values of the L/L interfacial tension and to achieve the negative value of Gibbs energy [9,22]. Both these systems are fundamentally different and behave differently in different applied conditions which is helpful in their identification. NE and ME are physicochemically different systems but their closely resembling terminologies, structural and formulation aspects, along with similar fabrication processes often lead to false identification of the system, sometimes, even based only on their droplet size [9]. The terminology used to describe these dispersions is also a subject of discussion as it appears from the terms NE and ME that the droplets in NE are smaller than those in ME, but in reality, the opposite is true [9,10,11,24]. The term “micro” implies that the ME droplets are in micro range but in actual they are below 100 nm in size. Similarly, term “nano” means that the droplets of NE are in nanometer, which is true as well because the size of NE droplets is usually below 300 nm [13]. Lack of familiarity with the physical behavior of such dispersions among pharmaceutical researchers and formulators lead to a well-established mix-up between these two systems in the literature [20,21,31]. More worryingly, the actual ME are characterized through the processes which can alter or even destroy these systems. Correct detection of dispersion’s nature is necessary for appropriate interpretation, characterization and correct target biological and pharmaceutical applications of the developed system (Figure 3). Therefore, it is important to differentiate between ME and NE and confirm the precise physical nature of generated colloidal dispersion after adding water phase to SEDDS.

#### 2.4.1. Droplet Size Distribution

**Figure 4:**
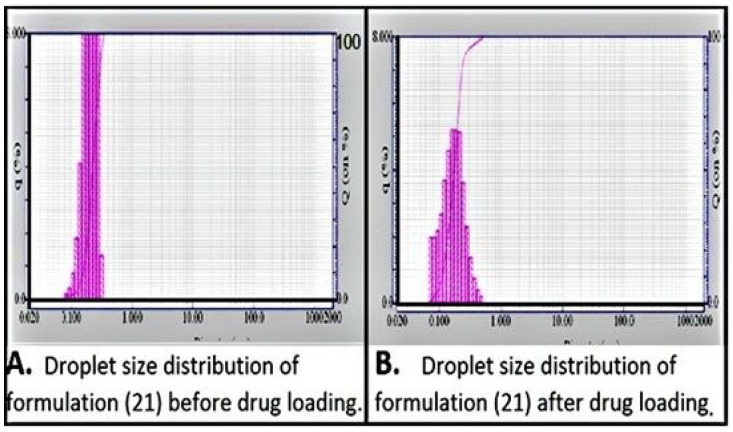
Graphs showing droplet size distribution of selected formulation where (**A**) Droplet size distribution of formulation 21 before drug loading and (**B**) Droplet size distribution of formulation 21 after drug loading.

The size and shape of the nano droplets is subjected to an intrinsic property of the surfactant used, i.e., its molecular arrangement at CMC [32,33,34]. Usually, amphiphiles arrange themselves in a way that they produce “minimum interfacial tension” and form micelles. At this point, the curvature is known as the “optimum curvature” and is specific for specific surfactants (due to specific molecular arrangements and hence, specific “minimum interfacial tensions”) [12,23]. The size and shape of the nano droplets in ME depends upon the surfactant and co-surfactant used along with their ratios. However, the nature of surfactant is more important as CMC plays the most important role in producing “minimum interfacial tension” and reaching “optimum curvature”. Therefore, the nature of ME is fundamentally identical to micelles, i.e., they are developed by amphiphiles at CMC and incorporate dispersed phase at their core. However, there are several differences between the micelles and microemulsions. For instance, the droplets in microemulsions contain much more dispersed liquid than in swollen micelles. Furthermore, whatever the size or shape of the nano droplets, they are constant throughout the dispersion. The reason for this is that intrinsic property of the surfactant is ought to remain same in the same dispersion. Results in Figure 4A,B showed a broad peak suggesting that the formulation is probably a NE. Figure 4A,B shows the droplet size before and after loading the drug, respectively. Drug incorporation into the dispersed phase inside the micelles resulted in increased size and enhanced particle size distribution. There is a very slight variation (mostly negligible) in the particle size due to slight variation in the amount of incorporated oil in microemulsion. Therefore, ME generally tend to produce narrow peak when analyzed for their “particle size distribution”, because all the micelles are nearly equal in size, whereas NE being “conventional nano emulsions” usually show a broad peak or multiple peaks because all the droplets are not of same size (Figure 4). Surfactant molecules do not arrange themselves freely at CMC as in the case of ME, rather they are forced to assemble in a hydrophilic or aqueous environment, hence giving rise to different sizes of nano droplets (size varying in a close range) [35].

#### 2.4.2. Droplet Shape

**Figure 5:**
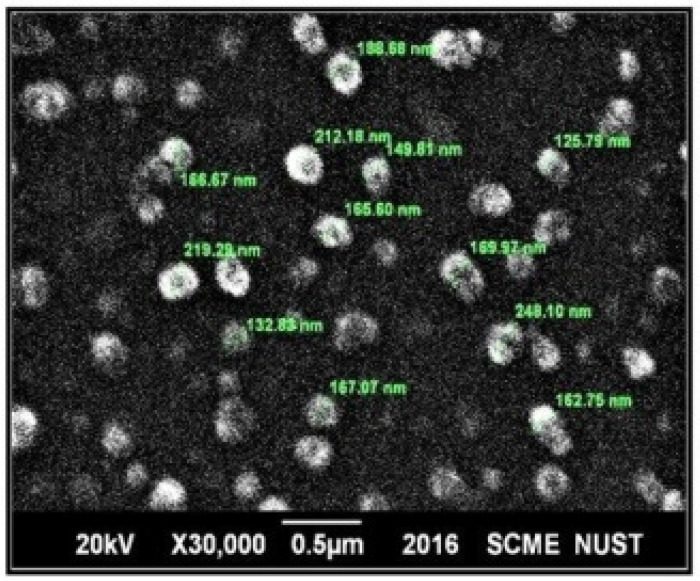
SEM image of regenerated liquid nanoemulsion developed from formulation (21).

As described earlier, the size and shape of the micelles in ME depend on the optimum curvature of the surfactant monolayer [36]. On the other hand, surfactant monolayers in NE have comparatively higher interfacial tensions which increases Laplace pressure. This pressure induces the spherical shape. Therefore, NE tend to have spherical shape droplets. Therefore, a dispersion with spherical shape droplets may be either NE or ME. However, if a system has non-spherical shape particles, it is probably a microemulsion [9,12]. Figure 5 depicts the droplets of the generated emulsion as spherical in shape. Therefore, it may be either a NE or a ME; however, it is less likely to be a ME.

#### 2.4.3. Dilution

Diluting the sample in the case of microemulsion can lead to a decrease in relative concentration of surfactant. As a consequence, the micelle size becomes smaller, as surfactant molecules try to reach the CMC. On further dilution, micelles and hence ME, might be destroyed due to the significant change in composition and decrease in surfactant concentration [11]. That is why diluting the samples for particle size analysis is meaningless. Dilution of ME not only makes characterization invalid by changing the size and shape of micelles, but can also destroy the ME, whereas dilution does not affect NE as they are sufficiently robust systems and can undergo dilution without rapid change in droplet size or destabilization [37]. Our results showed that SEDDS formulation is robust in dilution with no change in optical transparency, odor, droplet size and color, thus suggesting it is most likely to be NE.

#### 2.4.4. Temperature Change

As mentioned earlier, micelles are formed at specific composition (CMC) and specific conditions i.e., temperature and pressure, thus altering the thermodynamic variables e.g., temperature change can affect the microemulsion in two ways. First, it affects the solubility of surfactant and consequently its partition between oil and aqueous phases. Second, it influences and disturbs the equilibrium state of a thermodynamically stable formulation [9]. Therefore, varying temperature and composition results in modification of size, morphology and shape of the swollen micelles and even complete destabilization of the system. On the other hand, NE are not affected by the temperature change over a wide range of temperatures. Being fairly robust, NE can withstand temperature change as well as composition change without rapid destruction [24,37]. Dispersion of the optimized formulation was stable up to (82 °C) and showed that it is more likely to be a NE.

Returning to the same conditions after cooling, agitating or heating leads to regeneration of ME with same properties [12]. This is because ME are thermodynamically stable systems which require specific conditions (temperature and pressure) and specific composition. Additionally, ME are formed spontaneously. Therefore, in principle, if the imposed conditions are same before and after disturbing the system, then the regenerated ME should have the same properties as those of initial system. On the other hand, NE cannot achieve similar properties (e.g., particle size distribution, creaming, and overall microstructure) and may not be formed again at all [9,12]. The possible reason for this behavior is that properties of NE strongly depend upon the technique adopted for their fabrication [35]. Therefore, properties of the NE may be different when the system is returned to original conditions after disturbance. This might not be true in all cases e.g., if the NE has high kinetic stability or the ME has high energy barriers and slow process of mass transfer.

In our experiment, the dispersion was stable up to (82 °C) but became turbid on further heating. After cooling, the system did not return to its original condition and remained turbid. This experiment suggests that the dispersions are probably NE, and not the ME.

From the results of all these simple experiments, it was concluded that the dispersion generated by adding aqueous phase to optimized finasteride SEDDS, was a “NE”. Hence, the optimized formulation could be attributed as SNEDDS. After the identification of physical nature of the developed system, relevant and appropriate characterization techniques were used for further characterization of FDE loaded SNEDDS.

### 2.5. Characterization of Solid SNEDDS (S-SNEDDS)

#### 2.5.1. Physical Characterization of S-SNEDDS

The results in Table 3 showed the physical characteristics of two different S-SNEDDS powders (L-SNEDDS after being adsorbed on carrier powder). SNEDDS powder and tablets with Aerosil 200 as carrier showed better properties than those with Avicel as carrier. Therefore, Aerosil 200 was chosen as carrier and tablets with Aerosil 200 were selected as final FDE S-SNEDDS tablets.

#### 2.5.2. Surface Morphology

**Figure 6:**
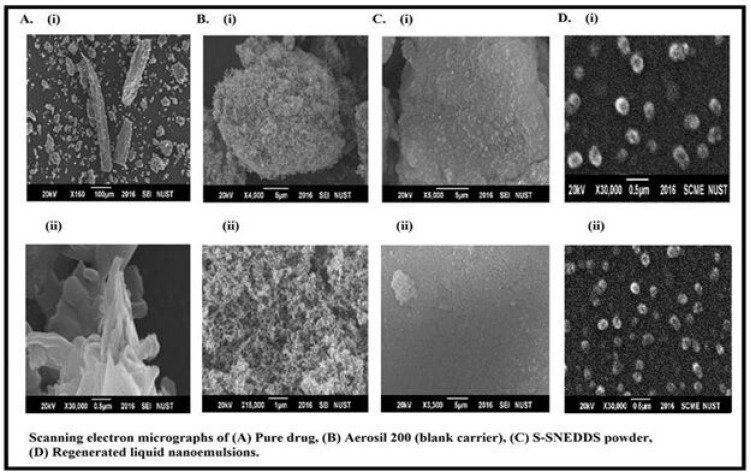
Scanning electron micrographs of (**A**) Pure drug, (**B**) Blank carrier, (**C**) Finasteride loaded S-SNEDDS powder and (**D**) Regenerated liquid nanoemulsion, whereas (**i**) and (**ii**) show respective SEM images at different magnifications.

Scanning electron micrographs (SEM) of pure FDE powder, aerosil 200, optimized S-SNEDDS and regenerated NE developed by the dilution of S-SNEDDS are shown in Figure 6A–D. FDE powder appeared as discrete and smooth surfaced crystals (Figure 6A) [38]. Amorphous particles of aerosil 200 with highly porous surfaces are evident in SEM photograph (Figure 6B). Whereas, optimized S-SNEDDS appeared as smooth surface particles without any crystalline shape indicating successful adsorption of amorphous FDE containing SNEDDS onto the surface pores of the carrier (Figure 6C). Regenerated dispersion showed spherical droplets which are not exactly equal in their size but lie in a close range of size. Figure 6D shows the successful development of NE from S-SNEDDS after dilution.

#### 2.5.3. Fourier Transform Infrared Spectroscopy

**Figure 7:**
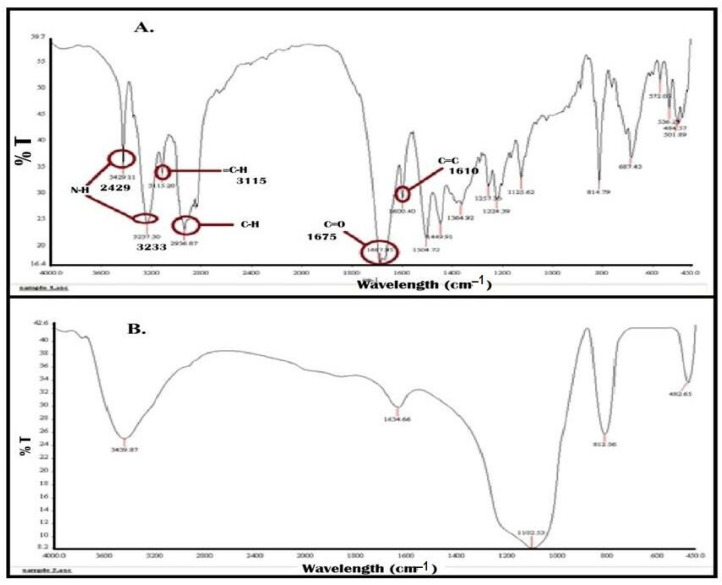
FTIR spectra of (**A**) Pure finasteride and (**B**) Aerosil 200 (blank carrier).

**Figure 8:**
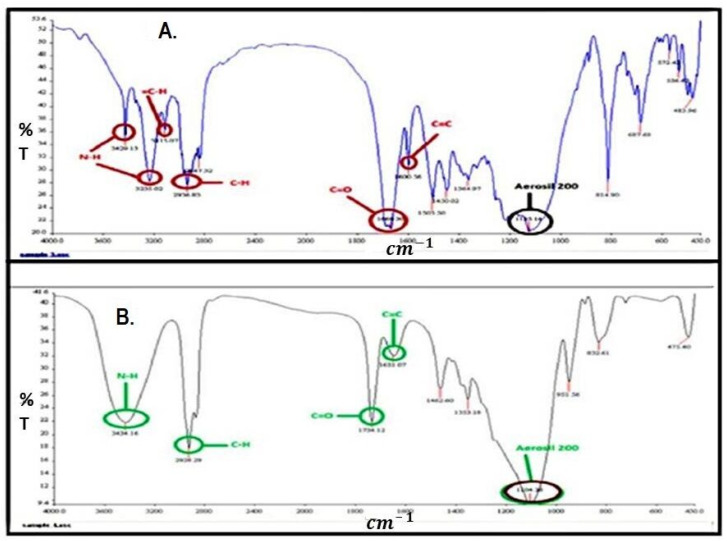
FTIR spectra of (**A**) Physical mixture of pure finasteride and aerosil 200 and (**B**) Finasteride S-SNEDDS powder.

The compatibility of the ingredients through FTIR was analyzed and results are shown in Figure 7 and Figure 8. Pure finasteride showed characteristic peaks at 1600 cm^−1^ (corresponding to C=C), 1687 cm^−1^ (C=O), 2936 cm^−1^ (-C-H), 3115 cm^−1^ (=C-H), 3237 cm^−1^ and 3429 cm^−1^ (N-H) (Figure 7A). All these characteristic peaks of FDE can be seen clearly in spectra of optimized S-SNEDDS (Figure 8B) indicating that the molecular structure of FDE was intact, and all the functional groups were present. There is no extra peak observed, which shows that there is no chemical interaction of drug with the excipients [39]. However, due to physical interaction, peak positions shifted slightly and showed some broadening effects.

#### 2.5.4. Powder X-ray Diffraction (PXRD) Studies

**Figure 9:**
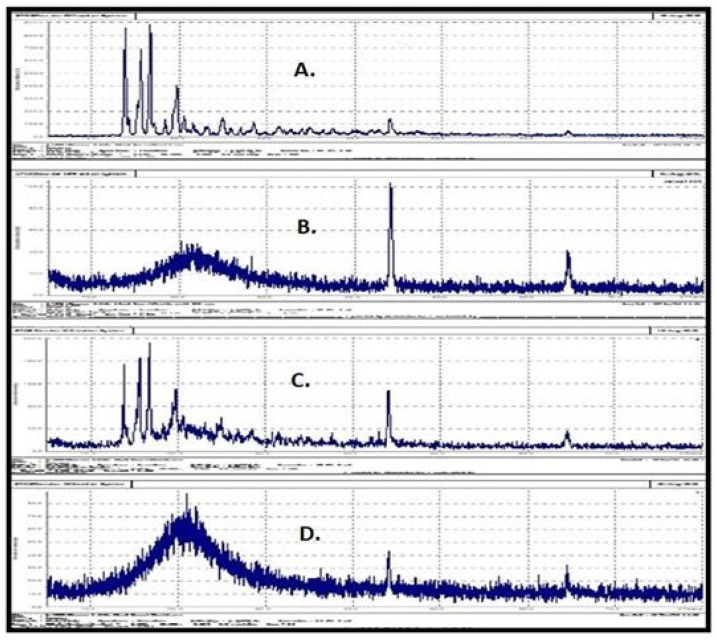
PXRD spectra of (**A**) Pure finasteride, (**B**) Aerosil 200 (blank carrier) (**C**) Physical mixture of finasteride and aerosil 200 and (**D**) Finasteride loaded S-SNEDDS powder.

The X-ray diffraction (XRD) studies are shown in Figure 9. Pure FDE showed characteristic sharp peaks at diffraction angles (Figure 9A). This indicates the crystalline nature of FDE. Figure 9B shows that aerosil 200 is present in amorphous form. XRD spectra of physical mixture of pure drug and aerosil 200 contained characteristic peaks of finasteride which implies that drug in the physical mixture is in crystalline form (Figure 9C), whereas optimized FDE loaded S-SNEDDS did not show sharp peaks of crystalline FDE, indicating that the drug is present in amorphous form in the formulation (Figure 9D). The amorphous form of the drug is beneficial as it minimizes the chances of in vivo precipitation by inhibiting the crystal formation [40,41].

#### 2.5.5. In Vitro Dispersion Profiles

**Figure 10:**
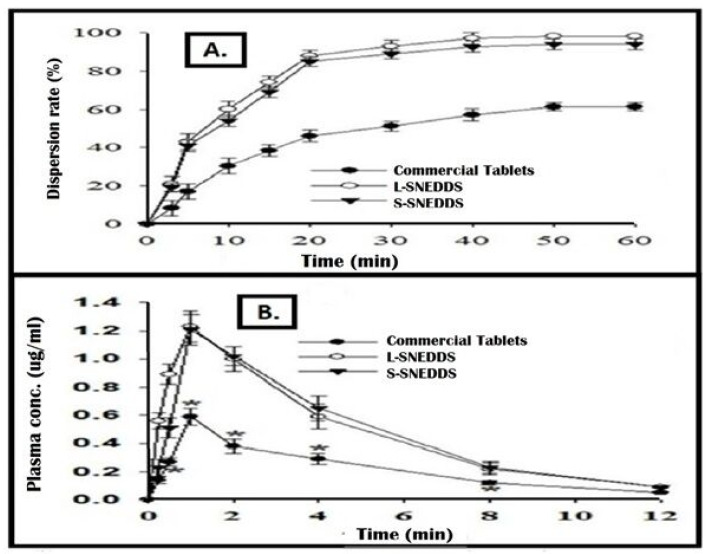
(**A**) In vitro dispersion profiles of S-SNEDDS tablets, L-SNEDDS and Genesis^®^ commercial tablets and (**B**) In vivo plasma drug concentrations at different time intervals. Each value represents the mean ± SD (n = 6).

In vitro dispersion profiles of liquid or L-SNEDDS and S-SNEDDS are compared with that of Genesis^®^ commercial tablets as shown in Figure 10A. It is obvious that FDE showed faster dispersion from L-SNEDDS as compared to S-SNEDDS; however, there is no significant difference. Nevertheless, both the L-SNEDDS and S-SNEDDS showed significantly enhanced dispersion as compared to the dissolution from commercial tablets. It is worth noting that the word “dispersion” has been used here instead of “dissolution” in the case of liquid or solid SNEDDS. This is because the drug is already dissolved in SNEDDS and only the dispersion phenomenon is happening here should be taken into account [29]. This is why the dispersion of drug from SNEDDS is faster than the dissolution of drug from the conventional tablets. As the dissolution step is skipped, SNEDDS can produce a quick absorption of the drug and a rapid onset of action [42]. Dispersion of NE droplets from S-SNEDDS is slightly slower than that from L-SNEDDS. This can be attributed to some additional steps involved before dispersion. These steps include disintegration of S-SNEDDS and release or desorption of L-SNEDDS from the porous carrier’s voids [43].

### 2.6. Drug Content

The theoretical content of finasteride in S-SNEDDS was 0.25% (145 mg carrier, 250 µL of L-SNEDDS weighing 250 mg, 5 mg drug in 400 mg total formulation), whereas the actual content evaluated was 0.24%. The actual to theoretical content ratio was found to be 96%, which is well within the limit.

### 2.7. Stability Studies

The stability of the formulation is an important parameter in establishing the shelf-life of the formulation. Stability studies of S-SNEDDS and L-SNEDDS were performed, and results are shown in Table 4. The results revealed no considerable change in drug content, droplet size, and in vitro release from S-SMEDDS up to six months. However, slight changes in droplet size and in vitro drug release were observed. The results concluded that S-SNEDDS are more stable as compared to L-SNEDDS.

### 2.8. In Vivo Bioavailability Study

Pharmacokinetic parameters were determined in order to obtain the bioavailability of finasteride from L-SNEDDS and S-SNEDDS and to compare with the commercial product, after oral administration to rats. The parameters are presented in Table 5 and Figure 9B. The results showed the plasma concentration of finasteride in rat blood samples at different time intervals. As represented, L-SNEDDS and S-SNEDDS showed significantly increased plasma concentration as compared to the commercial product. The L-SNEDDS and S-SNEDDS meaningfully improved the AUC 287.03 and 280.92 respectively, as compared to the commercial product (93.51). This shows that SNEDDS has the ability to enhance (three-fold) the bioavailability of the poorly water-soluble drug, finasteride. This ability could be explained on the basis of improved dissolution of the drug resulting from the small droplet size of regenerated NE. It is also worth mentioning that a burst release can be observed in L-SNEDDS, particularly in the first 30 min after their oral intake. This may lead to severe toxicity if used for clinical purposes. This is why we suggest S-SNEDDS as a better choice for obtaining maximum therapeutic effect with no toxicities. SNEDDS also provides the in vivo solubilization effect due to the presence of surfactant and co-surfactant. Improved bioavailability possibly may be because of the decreased enzymatic degradation, inhibition of P-gp efflux pumps, and enhanced absorption from the lipid bilayer due to the components present in the formulation i.e., capryol 90, cremophore RH 40, and PEG 400. Results of in vivo bioavailability suggest that it would be possible to formulate finasteride S-SNEDDS tablets with decreased therapeutic dose reducing the side effects and cost associated with the drug [9,29,44].

## 3. Materials and Methods

### 3.1. Materials

Finasteride was a kind gift from Ferozsons Laboratories limited, Pakistan. Labrafil M 1944 CS and M 2125 CS as long chain triglycerides (LCT), Capryol 90, Capryol PGMC and Labrafac lipophile WL 1349 as medium chain triglycerides (MCT) (Gattefosse, France). Soyabean oil, olive oil and coconut oil were purchased from local market. Triacetin as short chain triglyceride (SCT) (Alfa Aesar, Germany), Cremophore (RH 40), Tween-80 (Sigma Aldrich, Germany), PEG 400 (Bio-Labs, Islamabad). All the chemical and solvents used were of analytical grade.

### 3.2. Methods

#### 3.2.1. Solubility Studies

Saturation solubility of FDE in different oils (4 long chain triglycerides, 4 medium chain triglycerides, 1 short chain triglyceride), surfactants (Cremophore RH 40 and Tween 80) and co-surfactants (PEG 400 and Labrasol) was evaluated using shake flask method [29,30]. An excess amount of drug (200 mg) was added to 2 mL of each vehicle in different vials and vortexed for 5 min to form a homogenous mixture. All these mixtures were incubated at 37 °C for 48 h in a shaker water bath SV 1422, WNB 14, (Memmert, Germany) operating at 50 strokes/min. The resultant solutions were centrifuged at 6000 rpm for 10 min using Z-206A (Hermle labortechnik, Germany) centrifugation machine to separate the excess FDE.

#### 3.2.2. Selection of Surfactant and Co-Surfactant

Briefly, (0.5 g) of selected oil was added to the same weight of surfactant. The mixture was heated to 50 °C and vortexed for 5 min to ensure homogenization 50 mg of each resultant mixture was diluted in 50 mL of deionized water and emulsification time was noted. Each of the resultant fine emulsions was observed visually for turbidity, phase separation and clarity. Then these emulsions were equilibrated for 2 h and evaluated for their percentage transmittance by UV-visible spectrophotometer Halo DB-20 (Dynamica, UK) at 638.2 nm.

Similarly, a co-surfactant was selected using a similar method. The selected surfactant (0.3 g) was added to (0.2 g) of each co-surfactant. This mixture was then mixed with (0.5 g) of selected oil and the same protocol was followed as described earlier for the selection of surfactant.

#### 3.2.3. Construction of Ternary Phase Diagram

Self-emulsifying region was identified by constructing the ternary phase diagrams. The selected surfactant was mixed with screened co-surfactant in three different (1:1, 1:2, 1:3) weight ratios as shown in Table 6 [31]. The oil phase and Smix (surfactant and co-surfactant mixture) were mixed thoroughly in nine different (1:9, 2:8, 3:7, 4:6, 5:5, 6:4, 7:3, 8:2, 9:1) weight ratios with total of 27 ratios. Of these mixtures, 1 mL was diluted separately with 100 mL distilled water and agitated gently using magnetic bars at 37 °C. The resulting emulsions were checked for their transmittance against blank (distilled water) at 638.2 nm using UV-visible spectrophotometer Halo DB-20 (Dynamica, UK). These emulsions were then examined for their phase separation, coalescence of oil droplets and clarity on standing for 2 h. Good emulsions were clear, transparent without coalescence, with percentage transmittance > 85, whereas bad emulsions showed phase separation and coalescence with percentage transmittance at < 85 and >70 [31]. Systems with percentage transmittance < 70 and coalescence, phase separation, or turbidity were poor dispersions. Codes were developed to classify good, poor, and very poor dispersions which are as follows: Good emulsions with % transmittance > 95 = +++, Good emulsions with % transmittance > 90 = ++, Good emulsions with % transmittance > 85 = +, Poor emulsions with % transmittance < 85 and >70 = -, Very poor emulsions = --. Ternary phase diagrams were constructed using SigmaPlot^®^ 12.0 software and nanoemulsifying ratios of excipients were identified.

#### 3.2.4. Preparation of Liquid SEDDS (L-SEDDS)

After identifying the self-emulsifying region, SEDDS were prepared by mixing oil phase and Smix in selected ratios. Drug-loaded L-SEDDS were prepared by solubilizing drug in oil phase first and then mixing oil phase with Smix at 37 °C and stored at room temperature for further studies [31].

#### 3.2.5. Percentage Transmittance, Saturated Solubility of SEDDS, and Self-Emulsification Time Determination

0.1 mL aliquot of each of the selected systems was diluted with 100-fold distilled water and percentage transmittance was observed against blank (distilled water) at 638.2 nm using UV-visible spectrophotometer Halo DB-20 (Dynamica, UK) [32].

Saturated solubility of selected SEDDS was determined to be able to limit the amount of FDE loaded in optimized SEDDS. Excess amount of FDE was added into 2 mL of the selected systems separately in centrifugal tubes, vortexed for 5 min and mixed in shaking water bath (thermostatically controlled) at 100 rpm and 37 °C for 48 h. This mixture was centrifuged at 6000 rpm for 10 min and soluble FDE in supernatant was measured using HPLC [33].

Self-emulsification efficiency of finasteride SEDDS was evaluated with standard dissolution apparatus II DT-820 (Erweka, Germany) mL of finasteride L-SEDDS was added to 900 mlL distilled water, drop by drop, using a pipette with a paddle speed of 50 rpm at 37 °C. The time required to disperse and disappear the SEDDS uniformly was called the self-emulsification time [34].

#### 3.2.6. Optimization of L-SEDDS

Final L-SEDDS formulation was selected on the basis of percentage transmittance, saturated solubility, and self-emulsification time [35]. The optimized formulation was then subjected to further evaluations.

#### 3.2.7. Confirmation of Dispersion’s Nature (Distinction between Nanoemulsions and Microemulsions)

##### Particle Size Distribution, Polydispersity and Shape Analysis

0.5 mL of L-SNEDDS was diluted in 200 mL distilled water and then sonicated for 15 min. A particle size analyzer LA-920 (Horiba, Japan) was used to scan the particle size distribution and polydispersity index in the regenerated dispersion. The solvent was water with a refractive index of 1.3 and viscosity of 0.0 poise. The particle shape in the regenerated dispersion was observed with analytical scanning electron microscope JSM-6490 A (Jeol, Japan) [34].

##### Dilution and pH Change

1 mL of optimized formulation was diluted 50, 100 and 1000 times with pH 6.8 PBS, 0.1 M HCl and distilled water. After keeping for 24 h, diluted samples were examined for phase separation, coalescence, particle size and clarity. The pH of the sample diluted 100 times with water was checked and attributed as pH of finasteride SNEDDS [36].

##### Effect of Temperature Change

The temperature of regenerated emulsion increased slowly at a rate of 0.5 °C/min in a water bath and emulsion was observed closely for optical transparency and soon after appearance of cloudiness in it, emulsion was removed from water bath and cooled down. Physical properties were investigated again at room temperature [37].

##### Effect of Centrifugation

The formulations were subjected to vigorous spinning (6000 rpm for 30 min) and the signs of creaming, cracking, drug precipitation, and phase separation were observed visually [29].

#### 3.2.8. Preparation of Solid SEDDS (S-SEDDS)

Liquid formulations (L-SEDDS) were converted to solid SEDDS (S-SEDDS) using the simplest solidifying technique. L-SEDDS were adsorbed on the surface of inert solid carriers, aerosil 200 and Avicel. An amount of L-SEDDS equivalent to dose of finasteride (5 mg) was adsorbed on the surface of solid carriers separately. The carrier was added in increments to a China dish containing L-SEDDS until the formation of a free flow powder. This powder (dose equivalent) was compressed into a tablet using a Single punch tablet press (CSP 12, 13721, Liaoyang Pharma Machinery) [34].

#### 3.2.9. Characterization of S-SEDDS

##### Surface Morphology

Surface morphology of the pure drug, carrier, and optimized S-SEDDS was evaluated using analytical scanning electron microscope JSM-6490 A, (Jeol, Japan). Samples were placed on double sided carbon conductive tape on stub. Gold coating (250 Å) was carried out with Ion sputtering device JFC-1500, (Jeol, Japan) to make the surfaces conductive [38].

##### Fourier Transform Infrared Spectroscopy (FTIR)

FT-IR spectroscopy was carried out to evaluate the possible interactions [34]. FT-IR spectra of pure drug, aerosil 200, physical mixture, and optimized S-SEDDS formulation were recorded on FT-IR spectrophotometer Spectrum 100, (PerkinElmer, Waltham, MA, USA) using KBr (Potassium bromide) pallet technique and then scanned over a wave number region of (450–4000 cm^−1^).

##### Powder X-ray Diffraction (PXRD) Studies

Powder crystallographic pattern of pure finasteride, physical mixture (aerosil 200 and finasteride) and optimized finasteride S-SEDDS formulation was determined through powder X-ray diffraction studies using X-ray diffractometer θ-θ, (STOE, Germany). Monochromatic Cu radiation λ = 1.54060 Å at voltage 20 kV and generator current of 5 mA was used. The scan was recorded over a range of 2θ from 5 to 80 with scan step time of 0.5 s/step [38].

##### Physical Characterization of S-SEDDS

***a*** ***Analysis of flow properties S-SEDDS powder***

The flow properties were analyzed through angle of repose, Carr’s index, Hausner’s ratio using the standard protocols [33].

***b*** ***Hardness of S-SNEDDS tablets***

Crushing strength of the S-SEDDS tablets was evaluated with hardness tester TT BIIIE, Pharma Test, D-63512 (Hainburg, Germany) [33].

***c*** ***Friability testing S-SNEDDS tablets***

The friability evaluation was performed as per standard protocols, with the friabilator PT F10E, Pharma Test, D-63512 (Hainburg, Germany) [33].

***d*** ***Uniformity of weight and disintegration time S-SNEDDS tablets***

Weight of tablets was measured and expressed as mg ± S.D. Disintegration apparatus PT ZS, Pharma Test, D-63512, (Hainburg, Germany) was used to find out the disintegration time following official USP procedure.

***e*** **In vitro** ***dispersion profile***

In vitro dispersion profiles of Genesis^®^ commercial tablets, optimized L-SEDDS and S-SEDDS tablets were investigated using USP dissolution apparatus II DT-820 (Erweka, Germany). The dialysis bag method was used with a nominal molecular weight cut off 12,000-14,000 (CelluSep^®^). In vitro release tests were carried out in 900 mL distilled water as dissolution medium at 37 ± 0.5 °C with 50 rpm for 60 min as per USP guidelines. L-SEDDS equivalent to 5 mg FDE was filled in dialysis bag after diluting with 2 mL of dissolution medium. 1 mL sample was withdrawn after predetermined time intervals. Each time an equal volume of dissolution medium was added and sink conditions were maintained. Each sample was filtered through 0.45 µm membrane filter and subjected to finasteride quantification using HPLC method using below mentioned conditions. Finasteride concentration in the supernatant was determined using HPLC, equipped with a (4.6 mm × 5 cm) column and absorbance was taken at 220 nm detector. The sample flow rate was set at 2 mL/min with column temperature 45 °C. Injection volume was 200 µL and tailing factor was less than 2. Acetonitrile and distilled water (29:21) mixture was used as mobile phase. Acetonitrile and distilled water (7:3) solution was used as diluting solution [31].

***f*** ***Drug content***

Precisely weighed 100 mg of FDE loaded S-SEDDS was diluted with 100 mL of ethanol and then sonicated for 30 min at 35 °C to extract the finasteride. This solution was centrifuged for 20 min at 6000 rpm. Undissolved carrier was separated by filtering the supernatant with 0.45 µm membrane filter and then detected by HPLC described earlier. Following equation was employed to determine drug content.

(1) $$Drug\;Content\;\left(\%\right)=\frac{Drug\;\left(supernatant\right)}{Drug\;\left(Total\;amount\right)}\times 100$$

##### Stability Studies

Physical and chemical stability of finasteride S-SEDDS was evaluated as per ICH guidelines at 25 ± 2 °C/60 ± 5% RH and 40 ± 2 °C/75 ± 5% RH. S-SEDDS tablets and L-SEDDS filled in size (0) hard gelatin capsules were kept for three months in a stability chamber HPP 260 (Memmert, Germany). Developed S-SEDDS and L-SEDDS formulations were assessed for their transmittance, drug content, in vitro dispersion, droplet size, appearance, color, and phase separation after 1, 2, 3, and 6 months [34].

##### In Vivo Bioavailability Study

This study was performed with 18 healthy albino rats weighing 300–500 g following the approved protocol of Bio-Ethical Committee (BEC) of Quaid-i-Azam University (Protocol No. BEC-FBS-QAU2017-52) based on the U.K. Animals (Scientific Procedures) Act, 1986 and associated guidelines, EU Directive 2010/63/EU for animal experiments and National Institutes of Health guide for the care and use of Laboratory animals (NIH Publications No023, revised 1978). The rats were divided into three groups (n = 6) and were kept on a standard diet. The first group received marketed formulation, the second group received L-SEDDS, and the third group received S-SEDDS in an amount equivalent to the calculated finasteride dose (75 µg/kg body weight). Blood samples (0.3 mL) were collected from right femoral artery of each rat at predetermined intervals. A total of six samples were collected from each group as only one blood sample was collected from each rat. Blood samples were centrifuged at 9000 rpm for 10 min and plasma was transferred to separate tubes. The samples were then analyzed through HPLC method as discussed above. Win Nonlin software (professional version, edition 5.2; Pharsight, Mountain View, CA, USA) was used to calculate the various pharmacokinetic parameters [31,38,45,46,47,48,49,50].

##### Statistical Analysis

The experiments were performed in triplicates at least, and results are shown as mean ± S.D.

The results were subjected to statistical analysis using ANOVA and Student’s t-tests, keeping *p* < 0.05, through SPSS version 21.

## 4. Conclusions

The study presents, for the first time, the practical distinction between fundamentally different lipidic systems on the basis of their energy dynamics through inherent properties exploration. Results suggest the possibility of successful differentiation between thermodynamically stable (SMEDDS) and unstable (SNEDDS), which is compulsory for correct interpretation of their nature, appropriate characterization, better understanding of stability parameters, improvement in their pharmaceutical applications and reducing associated cost. The model study clearly defined the synthesis of nanoemulsion i.e., SNEDDS which were characterized using various in vitro and in vivo techniques. Size analysis revealed that droplet size was lesser than 180 nm. A novel evaluation “differentiation between microemulsions (fundamentally distinct from emulsions) and nanoemulsions (true emulsions)” confirmed that the dispersion developed was thermodynamically unstable nanoemulsion and not a microemulsion. S-SNEDDS and L-SNEDDS showed significantly increased in vitro dispersion of finasteride, 94% and 98%, respectively, as compared to the commercial tablets (61%) in one hr. Similarly, the in vivo study showed enhanced AUC and Tmax of S-SNEDDS and L-SNEDDS as compared to the commercial tablets, thus resulting in a three-fold improved bioavailability of the model drug. All these evaluated parameters suggested that solidified SNEDDS have superior potential to be developed as a drug carrier for oral delivery of lipophilic drugs such as finasteride. Therefore, the knowledge of dispersion’s precise physicochemical nature is inevitable for selecting their administration route, manufacturing process, characterization techniques, stability, use and pharmaceutical applications.

## Figures and Tables

**Table 1:** Finasteride solubilizing capacity and emulsification ability of surfactants and co-surfactants. The results are shown as mean ± S. D.

Surfactant	Solubility (mg/mL)	Self-Emulsification Time (s)		Percentage Transmittance (%)	
**Tween 80**	46 ± 6	50		80	
**Cremophore RH 40**	20 ± 5	20		99	
		**Cremophore RH 40**	**Tween 80**	**Cremophore RH 40**	**Tween 80**
**Co-surfactant** (used along with surfactants Cremophore RH 40 and Tween 80)					
**PEG 400** (Co-surfactant)	21 ± 3	10	38	99	84
**Labrasol** (Co-surfactant)	12 ± 2	16	42	98	84

**Table 2:** Physicochemical characterization of selected formulations. The results are shown as mean ± S.D.

Formulation with Composition (oil, Surfactant, Co-Surfactant)	Saturated Solubility (mg/mL)	Percentage Transmittance (%)	Droplet Size (nm)	Self-Emulsification Time (s)
13 (40%, 40%, 20%)	21	87	273	10
**21** **(30%, 52.5% 17.5%)**	**22**	**94**	**180**	**10**
22 (40%, 45%, 15%)	21	92	211	15

**Table 3:** Results of physical characterization tests for S-SNEDDS powders and S-SNEDDS tablets.

Ingredients	Formulation 1 (Aerosil 200)	Formulation 2 (Avicel)
	Formulations (% *w*/*w*)	
**Finasteride**	1.25	1.25
**Cremophore RH 40**	32	32
**Capryol 90**	18	18
**PEG 400**	11	11
**Avicel**	----	36.75
**Aerosil**	36.75	----
**Mg stearate**	1	1
**Carr’s index (CI)**	11%	6%
**Angle of repose**	25°	31°
**Hardness of tablets**	5.5 ± 0.35 Kg	4.2 ± 0.71 Kg
**Hausner ratio (HR)**	1.12	1.0
**Friability test**	0.30 ± 0.1%	0.45 ± 0.05%
**Disintegration time**	6 min	6 min
**Uniformity of weight** **(Tablets)**	400 ± 2.4 mg	400 ± 3.5 mg

**Table 4:** Results of stability studies of S-SNEDDS tablets and L-SNEDDS after 1, 2, 3 and 6 months at 40 ± 2 °C/75 ± 5% RH. The results are presented as mean ± SD.

Time (Month)	Droplet Size (nm)		Drug Content (%)		In Vitro Dispersion (% Drug Released after 60 min)	
	S-SNEDDS Tablets	L-SNEDDS	S-SNEDDS Tablets	L-SNEDDS	S-SNEDDS Tablets	L-SNEDDS
**1**	182 ± 2	190 ± 1	>95 ± 1.23	>95 ± 1.32	92% ± 0.56	93% ± 1.00
**2**	190 ± 1	195 ± 2	>95 ± 1.40	>95 ± 1.56	93% ± 1.41	90% ± 1.88
**3**	195 ± 1	193 ± 3	>95 ± 1.52	>90 ± 1.63	90% ± 1.51	84% ± 1.50
**6**	194 ± 2	210 ± 3	>95 ±1.50	>90 ± 2.71	93% ± 1.62	81% ± 2.19

**Table 5:** Pharmacokinetic parameters of finasteride loaded SNEDDS after oral administration to rats. The results are presented as mean ± SD. * *p* < 0.05 compared with the S-SNEDDS tablets and L-SNEDDS.

Formulation	AUC (h. µg/mL)	C_max_ (µg/mL)	T_max_ (h)	t_1/2_ (h)	K_e_ (h^−1^)
S-SNEDDS tablets	280.92 ± 33.9	1.214 ± 0.45	1.02 ± 0.2	9.92 ± 1.51	0.09 ± 0.02
L-SNEDDS	287.03 ± 34.4	1.236 ± 0.42	1.00 ± 0.2	9.01 ± 1.47	0.10 ± 0.02
Commercial tablets	93.51 ± 10.73 *	0.591 ± 0.21 *	1.12 ± 0.5	7.76 ± 1.16	0.19 ± 0.05

**Table 6:** Composition of different SNEDDS formulations (27) using different excipient ratios and results of physical examination with percentage transmittance. (Good emulsions with % transmittance > 95 = +++, Good emulsions with % transmittance > 90 = ++, Good emulsions with % transmittance > 85 = +, Bad emulsions with % transmittance < 85 and > 70 = -, Poor dispersions = --).

Title	Formulation Code with Percentage Transmittance and Results of Physical Examination in Codes						Oil Phase (Capryol 90) µL			Surfactant (Cremophore RH 40) µL			Co-Surfactant (PEG 400) µL		
Km Ratio (Surfactant to co-surfactant ratio)	1:1		2:1		3:1		1:1	2:1	3:1	1:1	2:1	3:1	1:1	2:1	3:1
	X 1	93 (++)	X 10	94.5 (++)	X 19	21.2 (--)	100	100	100	450	600	675	450	300	225
	X 2	94.5 (++)	X 11	94.6 (++)	X 20	95.1 (+++)	200	200	200	400	533	600	400	267	200
	X 3	94.5 (++)	X 12	94.6 (++)	X 21	96.6 (+++)	300	300	300	350	467	525	350	234	175
	X 4	93.4 (++)	X 13	92.4 (++)	X 22	96.1 (+++)	400	400	400	300	400	450	300	200	150
	X 5	94 (++)	X 14	92.5 (++)	X 23	93.4 (++)	500	500	500	250	333	375	250	167	125
	X 6	86 (+)	X 15	92.3 (++)	X 24	91 (++)	600	600	600	200	266	300	200	134	100
	X 7	72 (-)	X 16	84 (-)	X 25	91 (++)	700	700	700	150	200	225	150	100	75
	X 8	10 (--)	X 17	47 (--)	X 26	86 (+)	800	800	800	100	133	150	100	67	50
	X 9	01 (--)	X 18	25 (--)	X 27	71.4 (-)	900	900	900	50	66	75	50	34	25

## Data Availability

Data is contained within the article.

## References

[1] Fong S.Y.K., Martins S.M., Brandl M., Bauer-Brandl A. (2016). Solid Phospholipid Dispersions for Oral Delivery of Poorly Soluble Drugs: Investigation into Celecoxib Incorporation and Solubility-In Vitro Permeability Enhancement. J. Pharm. Sci..

[2] Rehman F.U., Shah K.U., Shah S.U., Khan I.U., Khan G.M., Khan A. (2016). From nanoemulsions to self-nanoemulsions, with recent advances in self-nanoemulsifying drug delivery systems (SNEDDS). Expert Opin. Drug Deliv..

[3] Yeom D.W., Song Y.S., Kim S.R., Lee S.G., Kang M.H., Lee S., Choi Y.W. (2015). Development and optimization of a self-microemulsifying drug delivery system for ator vastatin calcium by using D-optimal mixture design. Int. J. Nanomed..

[4] Singh G., Pai R.S. (2015). Trans-resveratrol self-nano-emulsifying drug delivery system (SNEDDS) with enhanced bioavailability potential: Optimization, pharmacokinetics and in situ single pass intestinal perfusion (SPIP) studies. Drug Deliv..

[5] Cherniakov I., Domb A.J., Hoffman A. (2015). Self-nano-emulsifying drug delivery systems: An update of the biopharmaceutical aspects. Expert Opin. Drug Deliv..

[6] Mir M., Ishtiaq S., Rabia S., Khatoon M., Zeb A., Khan G.M., Rehman A.U., Din F.U. (2017). Nanotechnology: From In Vivo Imaging System to Controlled Drug Delivery. Nanoscale Res. Lett..

[7] Wang Z., Sun J., Wang Y., Liu X., Liu Y., Fu Q., Meng P., He Z. (2010). Solid self-emulsifying nitrendipine pellets: Preparation and in vitro/in vivo evaluation. Int. J. Pharm..

[8] Shakeel F., Haq N., El-Badry M., Alanazi F.K., Alsarra I. (2013). Ultra fine super self-nanoemulsifying drug delivery system (SNEDDS) enhanced solubility and dissolution of indomethacin. J. Mol. Liq..

[9] Anton N., Vandamme T.F. (2010). Nano-emulsions and Micro-emulsions: Clarifications of the Critical Differences. Pharm. Res..

[10] Fanun M. (2008). Microemulsions: Properties and Applications.

[11] Wennerström H., Balogh J., Olsson U. (2006). Interfacial tensions in microemulsions. Colloids Surf. A Physicochem. Eng. Asp..

[12] McClements D.J. (2012). Nanoemulsions versus microemulsions: Terminology, differences, and similarities. Soft Matter.

[13] Solans C., Izquierdo P., Nolla J., Azemar N., Garcia-Celma M.J. (2005). Nano-emulsions. Curr. Opin. Colloid Interface Sci..

[14] McClements D.J. (2010). Edible nanoemulsions: Fabrication, properties, and functional performance. Soft Matter.

[15] McClements D.J. (2015). Food Emulsions: Principles, Practices, and Techniques.

[16] Jonsson B., Lindman B., Holmberg K., Kronberg B. (1998). Surfactants and Polymers in Aqueous Solution.

[17] Yang Y., Leser M.E., Sher A.A., McClements D.J. (2012). Formation and stability of emulsions using a natural small molecule surfactant: Quillaja saponin (Q-Naturale®). Food Hydrocoll..

[18] Yi T., Wan J., Xu H., Yang X. (2008). A new solid self-microemulsifying formulation prepared by spray-drying to improve the oral bioavailability of poorly water soluble drugs. Eur. J. Pharm. Biopharm..

[19] Zhang P., Liu Y., Feng N., Xu J. (2008). Preparation and evaluation of self-microemulsifying drug delivery system of oridonin. Int. J. Pharm..

[20] Ansari K.A., Pagar K.P., Anwar S., Vavia P.R., Storti-Filho A., Damke E., Carrara M.A., Batista M.R., Donatti L., Boer C.G. (2014). Design and optimization of self-microemulsifying drug delivery system (SMEDDS) of felodipine for chronotherapeutic application. Braz. J. Pharm. Sci..

[21] Fagir W., Hathout R.M., A Sammour O., ElShafeey A.H. (2015). Self-microemulsifying systems of Finasteride with enhanced oral bioavailability: Multivariate statistical evaluation, characterization, spray-drying and in vivo studies in human volunteers. Nanomedicine.

[22] Kahlweit M., Strey R., Busse G. (1990). Microemulsions: A qualitative thermodynamic approach. J. Phys. Chem..

[23] Kahlweit M., Busse G., Faulhaber B., Jen J. (1996). Shape Changes of Globules in Nonionic Microemulsions. J. Phys. Chem..

[24] Tadros T., Izquierdo P., Esquena J., Solans C. (2004). Formation and stability of nano-emulsions. Adv. Colloid Interface Sci..

[25] Da Silva L.M., Montanari C.M., Santos O.M.M., Cazedey E.C.L., Ângelo M.L., de Araújo M.B. (2015). Quality evaluation of the Finasteride polymorphic forms I and II in capsules. J. Pharm. Biomed. Anal..

[26] Cha E.K., Shariat S.F. (2011). The use of 5α-reductase inhibitors for the prevention and treatment of prostate cancer. Eur. Urol..

[27] Almeida H.M., Marques H.M.C. (2011). Physicochemical characterization of finasteride: PEG 6000 and finasteride: Kollidon K25 solid dispersions, and finasteride: β-cyclodextrin inclusion complexes. J. Incl. Phenom. Macrocycl. Chem..

[28] Zabkowski T. (2014). Evaluation of the clinical indications, adverse drug reactions, and finasteride use in patients with benign prostatic hyperplasia in Poland. Pharmacol. Rep..

[29] Bandopadhyay S., Katare O., Singh B. (2014). Development of optimized supersaturable self-nanoemulsifying systems of ezetimibe: Effect of polymers and efflux transporters. Expert Opin. Drug Deliv..

[30] Rashid R., Kim D.W., Din F.U., Mustapha O., Yousaf A., Park J.H., Kim J.O., Yong C.S., Choi H.-G. (2015). Effect of hydroxypropylcellulose and Tween 80 on physicochemical properties and bioavailability of ezetimibe-loaded solid dispersion. Carbohydr. Polym..

[31] Mohd A.B., Sanka K., Bandi S., Diwan P., Shastri N. (2013). Solid self-nanoemulsifying drug delivery system (S-SNEDDS) for oral delivery of glimepiride: Development and antidiabetic activity in albino rabbits. Drug Deliv..

[32] Gupta S., Chavhan S., Sawant K.K. (2011). Self-nanoemulsifying drug delivery system for adefovir dipivoxil: Design, characterization, in vitro and ex vivo evaluation. Colloids Surfaces A: Physicochem. Eng. Asp..

[33] Abdelbary G., Amin M., Salah S. (2013). Self nano-emulsifying simvastatin based tablets: Design and in vitro/in vivo evaluation. Pharm. Dev. Technol..

[34] Agrawal A.G., Kumar A., Gide P.S. (2014). Formulation of solid self-nanoemulsifying drug delivery systems using *N*-methyl pyrrolidone as cosolvent. Drug Dev. Ind. Pharm..

[35] Sinko P., Martin A. (2006). Micromeritics. J. Pharm. Pharm. Sci..

[36] Miao Y., Chen G., Ren L., Pingkai O. (2014). Characterization and evaluation of self-nanoemulsifying sustained-release pellet formulation of ziprasidone with enhanced bioavailability and no food effect. Drug Deliv..

[37] Baloch J., Sohail M.F., Sarwar H.S., Kiani M.H., Khan G.M., Jahan S., Rafay M., Chaudhry M.T., Yasinzai M., Shahnaz G. (2019). Self-Nanoemulsifying Drug Delivery System (SNEDDS) for Improved Oral Bioavailability of Chlorpromazine: In Vitro and In Vivo Evaluation. Medicina.

[38] Kang J.H., Oh D.H., Oh Y.-K., Yong C.S., Choi H.-G. (2011). Effects of solid carriers on the crystalline properties, dissolution and bioavailability of flurbiprofen in solid self-nanoemulsifying drug delivery system (solid SNEDDS). Eur. J. Pharm. Biopharm..

[39] Chen Y., Li G., Wu X., Chen Z., Hang J., Qin B., Chen S., Wang R. (2008). Self-Microemulsifying Drug Delivery System (SMEDDS) of Vinpocetine: Formulation Development and in Vivo Assessment. Biol. Pharm. Bull..

[40] Balakrishnan P., Lee B.-J., Oh D.H., Kim J.O., Hong M.J., Jee J.-P., Kim J.A., Yoo B.K., Woo J.S., Yong C.S. (2009). Enhanced oral bioavailability of dexibuprofen by a novel solid Self-emulsifying drug delivery system (SEDDS). Eur. J. Pharm. Biopharm..

[41] Rao J., McClements D.J. (2011). Formation of Flavor Oil Microemulsions, Nanoemulsions and Emulsions: Influence of Composition and Preparation Method. J. Agric. Food Chem..

[42] Evilevitch A., Jönsson B., Olsson U., Wennerström H. (2001). Molecular Transport in a Nonequilibrium Droplet Microemulsion System. Langmuir.

[43] Rao J., McClements D.J. (2012). Lemon oil solubilization in mixed surfactant solutions: Rationalizing microemulsion & nanoemulsion formation. Food Hydrocoll..

[44] Kahlweit M., Strey R., Busse G. (1993). Weakly to strongly structured mixtures. Phys. Rev. E.

[45] Izquierdo P., Esquena J., Tadros T.F., Dederen C., Garcia M.J., Azemar N., Solans C. (2001). Formation and Stability of Nano-Emulsions Prepared Using the Phase Inversion Temperature Method. Langmuir.

[46] Thi T.D., Van Speybroeck M., Barillaro V., Martens J., Annaert P., Augustijns P., Van Humbeeck J., Vermant J., Mooter G.V.D. (2009). Formulate-ability of ten compounds with different physicochemical profiles in SMEDDS. Eur. J. Pharm. Sci..

[47] Agrawal A.G., Kumar A., Gide P.S. (2014). Formulation development and in vivo hepatoprotective activity of self nanoemulsifying drug delivery system of antioxidant coenzyme Q10. Arch. Pharmacal. Res..

[48] Parmar K., Patel J., Sheth N. (2015). Self nano-emulsifying drug delivery system for Embelin: Design, characterization and in-vitro studies. Asian J. Pharm. Sci..

[49] Khan A.W., Kotta S., Ansari S.H., Sharma R.K., Ali J. (2012). Potentials and challenges in self-nanoemulsifying drug delivery systems. Expert Opin. Drug Deliv..

[50] Wang L., Dong J., Chen J., Eastoe J., Li X. (2008). Design and optimization of a new self-nanoemulsifying drug delivery system. J. Colloid Interface Sci..

